# Assessment of Acrylamide Levels by Advanced Molecularly Imprinted Polymer-Imprinted Surface Plasmon Resonance (SPR) Sensor Technology and Sensory Quality in Homemade Fried Potatoes

**DOI:** 10.3390/foods13182927

**Published:** 2024-09-15

**Authors:** Betül Karslıoğlu, Bahar Bankoğlu Yola, İlknur Polat, Harun Yiğit Alkan, Mehmet Lütfi Yola

**Affiliations:** 1 Department of Gastronomy and Culinary Arts, Faculty of Tourism, Hasan Kalyoncu University, Gaziantep 27000, Turkey; hyigit.alkan@hku.edu.tr; 2 Department of Engineering Basic Sciences, Faculty of Engineering and Natural Sciences, Gaziantep Islam Science and Technology University, Gaziantep 27010, Turkey; bahar.bankogluyola@gibtu.edu.tr; 3 Department of Nutrition and Dietetics, Faculty of Health Sciences, Hasan Kalyoncu University, Gaziantep 27000, Turkey; ilknur.polat@hku.edu.tr (İ.P.); mlutfi.yola@hku.edu.tr (M.L.Y.)

**Keywords:** acrylamide, sensory analysis, SPR, MIP, homemade fried potatoes

## Abstract

This study evaluated acrylamide (AA) levels and various quality parameters in homemade fried potatoes prepared in different sizes by integrating principles from the Slow Food Movement with advanced sensor technology. To this aim, a surface plasmon resonance (SPR) sensor based on a molecularly imprinted polymer (MIP) was first developed for the determination of AA in homemade fried potatoes at low levels, and the AA levels in the samples were established. First of all, monolayer formation of allyl mercaptane on the SPR chip surface was carried out to form double bonds that could polymerize on the chip surface. AA-imprinted SPR chip surfaces modified with allyl mercaptane were prepared via UV polymerization using ethylene glycol dimethacrylate (EGDMA) as a cross-linker, N,N′-azobisisobutyronitrile (AIBN) as an initiator, and methacryloylamidoglutamicacid (MAGA) as a monomer. The prepared AA-imprinted and nonimprinted surfaces were characterized by atomic force microscopy (AFM) and Fourier transform infrared (FTIR) spectroscopy methods. The SPR sensor indicated linearity in the range of 1.0 × 10^−9^–5.0 × 10^−8^ M with a detection limit (LOD) of 3.0 × 10^−10^ M in homemade fried potatoes, and the SPR sensor demonstrated high selectivity and repeatability in terms of AA detection. Additionally, the highest AA level was observed in the potato sample belonging to the T1 group, at 15.37 nM (*p* < 0.05), and a strong and positive correlation was found between AA levels and sensory parameters, the a* value, the ΔE value, and the browning index (BI) (*p* < 0.05).

## 1. Introduction

Recently, the “Slow Food Movement” has emerged as a global food trend [1,2]. The core philosophy of this movement, which impacts the entire sector, is clean, healthy, and fair food. It is an innovative and emerging food concept that prioritizes sustainability and encourages the consumption of nutritious foods at every stage, from production to consumption [3]. This ideology aims to support local producers, promote sustainable farming practices, and offer consumers healthier and tastier food options [4]. The term “slow food” is seen to overlap with many other specific concepts, such as “local production”, “health”, “environmental protection”, and “simplicity” [5]. Although Turkish cuisine adopted potatoes relatively late, they have become foods of historical and cultural importance. Researchers have also reported that potatoes are used in various dishes using different cooking techniques in Turkish cuisine [6,7]. These dishes include potatoes prepared using techniques such as roasting, frying, boiling, and roasting, with frying being the most commonly used cooking method. Frying potatoes is especially widely used in many local cuisines throughout Turkey [6]. However, during the frying process, chemical changes occur due to heat-induced processes such as Maillard and caramelization reactions [8,9]. While these reactions impart characteristic sensory properties to the food, they also lead to the formation of some undesirable compounds [9,10,11]. 

AA has been reported as one of the most undesirable compounds in starch-based foods due to its toxicity and carcinogenic properties [12,13]. This has led to extensive research on topics such as the mechanisms of AA formation, its presence in different foods, strategies to reduce its levels, and methods for its analytical detection [14,15,16,17,18,19,20]. This compound has also been reported in foods at above 120 °C, especially in starch-based products such as potato chips, French fries, bread, and cookies [21,22,23]. This compound is essentially produced by a series of non-enzymatic reactions in foods at high temperatures and in low-humidity environments [21,24]. The World Health Organization and the International Agency for Research on Cancer have classified AA as “probably carcinogenic to humans” (Group 2A) [14,20,25,26,27,28,29]. Numerous studies have investigated the levels of AA in various foods, including bread, coffee, and biscuits, as well as traditional and street foods [30,31,32]. Among these food items, potato products were reported to have the highest levels (150 and 4000 μg/kg) of this compound [22,33,34,35,36,37]. 

Potato products are susceptible to AA formation owing to the significant presence of precursors in the tubers and the intense thermal processing conditions they undergo [38]. Therefore, given the potential health risks associated with the high consumption rates of AA-containing French fries, it is imperative to develop fast, specific, convenient, and sensitive methods for detecting and monitoring the presence of this compound. In this context, AA determination is performed using mass spectrometric detection, along with separation methods such as high-performance liquid chromatography or gas chromatography [39,40,41]. However, the major challenge associated with these methods is that they are time-consuming, expensive, and intricate. Therefore, these difficulties have encouraged researchers to develop new methods for detection using alternative analytical approaches. Researchers have recently recommended SPR sensors for AA detection due to their advantages of being rapid, simple, and having higher sensitivity [42,43]. SPR is an optical sensor technology that measures changes in the local refractive index to evaluate molecular binding on a metal surface. SPR sensors utilize the phenomenon of surface plasmons to detect molecular interactions occurring at the sensor surface. These sensors have a rapid response time and are capable of the real-time monitoring of biomolecular interactions without the need for labels. SPR sensors allow for sample preparation, chemical analysis, and data evaluation simultaneously. Additionally, they enable the simultaneous analysis of binding and dissociation events in SPR sensors based on molecularly imprinted polymers (MIPs) [44,45,46]. 

The goal of the molecular imprinting technique is to develop selective materials with specific chemical functions through the interaction, either covalent or non-covalent, of functional monomers [47,48]. The molecular imprinting process includes the steps of pre-complexation, polymerization, and removal of the template molecule. Therefore, specific polymeric cavities tailored to the analyte molecule are formed. MIPs, frequently employed in separation and sensor applications, are structures resistant to high pressure, temperature, and physical stress [49,50], and they also possess an inert structure [47,51]. In the literature, various sensor techniques based on MIPs have been presented for AA determination. For instance, a photoelectrochemical sensor based on ZnO/polypyrrole nanocomposites was developed and applied to potato chips and biscuit samples. As a result, 1.0 × 10^−1^–2.5 × 10^−9^ M with a LOD of 2.15 × 10^−9^ M was observed [52]. In addition, MIPs based on Fe_3_O_4_ were prepared for AA enrichment, and the adsorption quantity was obtained at 19.28 mg g^−1^ [53]. 

Considering the consumption rates of fried potatoes in Turkish cuisine, the potential formation of AA, which poses health risks and threatens food safety, is one of the significant risks in the food sector. Therefore, it is important to determine the AA levels in different sizes and shapes of homemade fried potatoes in an accurate, fast, and simple way. In conclusion, one of the aims of this study, considering the increasingly popular Slow Food Movement in recent years, is to produce homemade fried potatoes that do not harm human health or the environment, using local ingredients sourced from local farmers. Another important aim of the study is to develop a fast, simple, and environmentally friendly sensor that will determine the AA levels in homemade fried potatoes prepared in different sizes and reveal the AA levels.

## 2. Material and Methods

### 2.1. Materials, Chemicals, and Apparatus

The potatoes and olive oil used in the study were sourced from local producers in Gaziantep as part of the Slow Food Movement. The raw material used in this study consisted of *Solanum tuberosum* L. potatoes of the Colomba variety, purchased from a local farmer in İzmir, Turkey. Extra virgin olive oil was supplied from Komili Co. in Gaziantep, Turkey. AA (CAS No: 79-06-1), methacrylamide (MA, CAS No: 71938-39-1), propionic acid (PA, CAS No: 79-09-4), DL-alanine (DL-ALA, CAS No: 302-72-7), L-asparagine (L-ASP, CAS No: 70-47-3), allyl mercaptane (CAS No: 90 870-23-5), MAGA, EGDMA (CAS No: 97-90-5), 2-hydroxyethylmethacrylate (HEMA, CAS No: 25249-16-5), AIBN (CAS No: 78-67-1), phosphate buffer solution (PBS, CAS No: 12352207), sodium chloride (NaCl, CAS No: 10378-23-1), potassium hexacyanoferrate (CAS No: 13746-66-2), zinc sulfate (CAS No: 7446-19-7), and hexane (CAS No: 95 110-54-3) were purchased from Sigma-Aldrich Merck Group Company (St. Louis, MO, USA). 

A Bruker-Tensor 27 FTIR spectrometer (Tokyo, Japan) and an AFM Park NX10 (Tokyo, Japan) were utilized for the characterization studies. Finally, the GenOptics SPR system from Calgary, AB, Canada, was used for analytical applications, and XanTec bioanalytics provided SPR chips (Duesseldorf, Germany).

In this study, statistical analyses were conducted on the data (Version 22.0, Minitab Inc., Enterprise Drive State College, PA, USA). Homemade fried potatoes were produced in two separate replications, with all analyzed parameters assessed in duplicate. To determine the differences between fried potatoes of various sizes, analysis of variance (ANOVA) was used, and Tukey’s post-hoc test was applied when the *p*-value was less than 0.05. Additionally, Pearson correlation analysis was conducted to evaluate the relationships between variables. All results in the study were presented as mean ± standard error.

### 2.2. SPR Chip Modification with Allyl Mercaptane

SPR chips were cleaned with acidic piranha solution H_2_SO_4_:H_2_O_2_ (3:1, 25.0 mL *v/v*) before modification. Following this process, the SPR chips were immersed in the solution and maintained in a shaking bath for 15 min. The shaking bath is laboratory equipment made from a container filled with heated water. After washing treatment with ultra-pure water several times, SPR chips were dried in an atmospheric nitrogen environment. For the modification of the vinyl groups required for the polymerization process on the chip surface, 3.0 M allyl mercaptane solution was dropped onto the clean SPR chips, and the self-assembling monolayer modification process was performed for 36 h. After washing treatment with ultra-pure water several times, the modified SPR chips were dried and preserved in an atmospheric nitrogen environment.

### 2.3. AA-Imprinted SPR Chip Preparation

Firstly, the MAGA-AA complex at a 2:1 molar ratio in the presence of PBS (2.0 mL, pH 6.0) was prepared under strong stirring for 1 h. After the transfer of the solution, including AIBN (2.0 mg), HEMA (1.0 mL), and EGDMA (1.0 mL), into the complex solution, the resultant dispersion as polymerization solution was interacted with nitrogen gas to provide an inert environment. After dropping the prepared above dispersion (100.0 µL) on the clean SPR chips via the spin-coating method with a rotation speed of 6000 rpm for a duration time of 2 min at 25 °C, UV polymerization was carried out for 20 min at 25 °C. The prepared SPR chip was tagged as MIP/SPR chips. AA non-imprinted SPR chips were prepared without the AA molecule (NIP/SPR) using the same procedure.

### 2.4. AA Removal from MIP/SPR Chips and Analysis Process

An amount of 1.0 M NaCl of the desorption agent was selected to break the electrostatic/hydrogen bond interactions. For this, AA-imprinted SPR chips were immersed into a 1.0 M NaCl solution (20.0 mL) under magnetic stirring for 20 min. After 20 min, the SPR chip was dried at 25 °C under nitrogen gas. 

After MIP/SPR chip placement into the SPR cell, PBS (2.0 mL, pH 6.0) was first passed for 10 min at a 1.0 mL min^−1^ flow rate to equilibrate the SPR system. Then, each AA adsorption solution (from 1.0 nM to 50.0 nM) was passed on the MIP/SPR chip for 40 min with a 1.0 mL min^−1^ flow rate until a constant resonance. Finally, the desorption process was completed using 1.0 M NaCl solution for 10 min. These steps of adsorption/desorption/regeneration were repeated for each AA amount.

### 2.5. Cooking Condition of Homemade Fried Potatoes and the Procedure of AA Analysis

In the study, potato samples were prepared using three different cutting methods, namely T1 (rondel), T2 (large dice), T3 (medium dice), and T4 (wedge), due to their impact on AA formation in fried potatoes. The raw potatoes to be used in the study were stored in a dark thermostat room at 4 °C and at 70.0% relative humidity until use. The homemade fried potatoes were prepared under conditions determined through preliminary trials. Potato tubers (medium-sized tubers) were washed with tap water, cleaned, and then peeled. Afterwards, the peeled potatoes were cut into T1 (rondel-50 × 3 mm), T2 (large dice-20 × 20 × 20 mm), T3 (medium dice-12 × 12 × 12 mm), and T4 (wedge-70 × 10 × 10 mm) sizes. Next, the potato samples were then soaked in water containing 1.0% NaCl for 15 min to remove starch and then dried to remove surface moisture. Finally, each dried potato sample was fried separately in a pot (1.0 L of oil). When the oil reached an initial temperature of 180.0 ± 0.5 °C, 200.0 g of potatoes were fried. The frying temperature and cooking time were measured during the frying of the potato samples. The potato samples were fried in a deep pan at 180.0 ± 0.5 °C for the following durations: T1 for 11 min, T2 for 15 min, T3 for 13 min, and T4 for 14 min. After frying, the samples were filtered on a wire sieve for 5 min and allowed to cool at 20 °C. Except for color and sensory analyses, all samples were homogenized using a blender and stored at 4 °C until analysis. In this study, all experiments were run in duplicate.

The samples were extracted using the method established by Verma and Yadav [54]. The fat of the finely ground potato samples was removed with hexane (repeated again two times), and 1.0 g was weighed into a 15.0 mL centrifuge tube. Then, 10.0 mL of ultra-pure water was added and centrifuged at 10 °C and 10,000 rpm for 10 min. After centrifugation, the filtrate was transferred to another centrifuge tube, and 0.5 mL of Carrez I and Carrez II solutions were added, respectively, and centrifuged again at 10.0 °C at 10,000 rpm for 10 min. Carrez I solution was prepared by dissolving 15.0 g of potassium hexacyanoferrate in 100.0 mL of water, and Carrez II solution was prepared by dissolving 30.0 g of zinc sulfate in 100.0 mL of water. After centrifugation, the remaining filtrate was kept in a hot water bath at 40 °C for 15 min to evaporate the water in the filtrate, and then it was filtered with 0.45 μm cellulose acetate syringe filter paper. Finally, the filtrates were diluted with PBS (pH 6.0) to fall within the prepared linearity range for the SPR sensor and were made ready for AA analysis.

### 2.6. Chemical and Physicochemical Parameters of Homemade Fried Potatoes

The moisture (950.46), fat (991.36), protein (955.04), and ash (920.153) contents of the samples were analyzed following the methods outlined by the Association of Official Analytical Chemists (AOAC) [55]. The pH was measured by dipping a pH electrode into a suspension of 10 g of potato samples homogenized with 100 mL of distilled water for one minute using a homogenizer (Hanna HI 221, Ann Arbor, MI, USA). In addition, the carbohydrate content was calculated using the following formula:

Carbohydrate content (%) = 100 − (Moisture(%) + Protein(%) + Fat(%) + Ash(%))

### 2.7. Sensory Properties in Homemade Fried Potatoes

Each homemade fried potato’s sensory evaluations were conducted between 2:30 PM and 3:30 PM. Panelists were selected from students and staff in the field of food science, and the panel consisted of 16 members, aged 18–41 years (5 female and 12 male), all from Hasan Kalyoncu University (Gaziantep, Turkey). Before the panel, all participants were asked to sign a written and verbal informed consent form to participate in the sensory tests. Panelists were asked to rate homemade fried potatoes of different sizes based on the intensities of the organoleptic properties: uniformity of blush, crispness, mealiness, oiliness, taste, and color (1 = extremely low intensity, 2 = low intensity, 3 = regular intensity, 4 = high intensity, 5 = extremely high intensity) [33]. Additionally, panelists were asked to rate the general acceptability of the homemade fried potatoes using a 5-point hedonic scale (1 = ‘extremely dislike’, 5 = ‘neither like nor dislike’, and 9 = ‘extremely like’) [56]. Potato samples were deep-fried and immediately presented to the panelists. The sample temperature was 75.0 ± 1.0 °C. In addition, the panelists were given samples in random numbers.

### 2.8. Color Evaluation

The surface color of the homemade fried potatoes of different sizes was determined using a Colorimeter CR-300 (Konica Minolta, Osaka, Japan). The L* value of the samples was used to indicate lightness, the a* value to indicate redness, and the b* value to indicate yellowness. The experiment was at room temperature, with each potato sample being taken from five different points. Additionally, the total color differences (ΔE) and browning index (BI) values of the fried potato samples were calculated using Equations (1) and (2) below [54,57]:

(1) $$\Delta E=\sqrt{\left\{{\left(a^{*}\right)}^{2}+{\left(b^{*}\right)}^{2}+{\left(L^{*}\right)}^{2}\right\}}$$

(2) BI=100(x−0.31)/0.172

where

$$x=(a^{*}+1.75\;L^{*})/(5.645\;L^{*}+a^{*}-3.012\;b^{*})$$

## 3. Results and Discussion

### 3.1. Chemical Composition of Homemade Fried Potatoes

The raw potato used in this study had an initial chemical composition of 18.83% dry matter, 12.77% protein, 0.23% fat, 4.55% ash (wet basis), and pH 6.5. The chemical composition and pH value results of fried potatoes of different sizes are summarized in Table 1. Significant differences were found between the moisture contents of different sizes of fried potatoes, with values ranging from 21.84% to 55.31% (*p* < 0.05). The highest amount of moisture was determined for T4, followed by T2, T3, and T1, at 55.08%, 47.90%, and 21.84%, respectively. According to these results, T1 had the highest moisture loss (59.33%), while T4 had the lowest moisture loss (25.86%).

The fat content of fried potatoes varies significantly depending on the frying conditions and the size and characteristics of the potatoes. The fat content in homemade fried potatoes of different sizes varied between 28.15% and 30.18% (*p* < 0.05). When examining the oil absorption percentage of fried potatoes, the lowest oil uptake rate was observed in the T1 group at 28.15%, while the highest rate was observed in the T4 group at 30.18%. According to the literature, oil absorption during frying may be linked to the amount of moisture that evaporates. As water and other compounds exit the potatoes during frying, oil is absorbed in return. Consequently, potatoes that lose less moisture tend to absorb less oil, as the reduced water release results in lower oil uptake by the product [58,59,60]. Conversely, as the moisture content of the product decreases, the amount of AA produced at a given temperature increases [61]. These findings are consistent with data reported by other researchers [58]. A study examining the impact of slice thickness on the oil uptake properties of French fries found that oil uptake increased with larger slice thickness [62]. 

### 3.2. Color Attributes of Homemade Fried Potatoes

The main reaction contributing to the color formation in fried potatoes is the Maillard reaction [63]. Therefore, determining the color parameters in fried potatoes is crucial, as it can serve as an indicator for AA levels and consumer acceptance. Table 2 shows the color measurement data for fried potato samples of various sizes. In this study, it was found that the L* value of fried potatoes prepared in different sizes ranged from 51.09 to 69.01, the a* value ranged from 3.94 to 11.78, and the b* value ranged from 33.58 to 36.02. 

Preparing fried potatoes in different sizes resulted in significant differences, especially in L* and a* values (*p* < 0.05). It was observed that in thinner and smaller potato samples (T1 and T3 groups), the frying process increased the a* value and resulted in a lower L* value (*p* < 0.05). Additionally, in fried potatoes, the browning index (BI), another parameter used to measure the extent of color change due to the Maillard reaction, was observed to be highest in the T1 group and lowest in the T4 group (*p* < 0.05). 

The ΔE value indicates the color difference between fresh potatoes and fried potato samples prepared in different sizes. Lower ΔE values suggest that the fried samples are closer in color to fresh potatoes. Our findings show that the size of the fries significantly affects the color parameters, with notable color changes observed between fries of different sizes and fresh potatoes. Specifically, the ΔE values measured in this study reveal that the size of the potatoes affects color changes, with significant differences observed between the T1 and T4 groups (*p* < 0.05). Our study found similar results to those reported in the study, showing that the size of the potato strips significantly affected the color parameters [64]. Comparable color results have also been documented in commercial French fries sold in local markets in India [54] and in fried potato strips [63]. 

### 3.3. Sensory Properties of the Homemade Fried Potatoes

The sensory analysis, which is a crucial factor in assessing the quality of fried potato strips, demonstrated the significant impact of size on various sensory parameters [65,66]. The evaluation focused on four different sizes and shapes of fried potato samples: 50 × 3 mm, 20 × 20 × 20 mm, 12 × 12 × 12 mm, and 70 × 10 × 10 mm, as detailed in Table 3. 

In this study, all sensory parameters were affected by the different sizes and shapes. (*p* < 0.05). The results indicate that preparing fried potatoes in different sizes improved characteristics such as color uniformity, crispiness, mealiness, oiliness, taste, and color. Among the different sizes, the 50 × 3 mm slices (T1 group) generally showed superior performance in overall acceptability, taste, blush, crispiness, oiliness, and color, as evidenced by higher sensory scores (*p* < 0.05). This suggests that the 50 × 3 mm size provides a better balance of sensory attributes, which likely contributes to its higher overall acceptability. According to the literature, color is the primary quality attribute assessed by consumers and plays a crucial role in product acceptance [62,67]. Consequently, the T1 group provided a more attractive color compared to the other groups (*p* < 0.05). 

In contrast, the T2 group (20 × 20 × 20 mm) received lower scores across many evaluated parameters, highlighting that larger sizes may not perform as well in terms of sensory quality. This could be due to the increased oil absorption and less favorable textural properties associated with larger slice sizes, which might affect their crispiness and overall sensory appeal. 

The study’s findings align with previous research, which found that thinner slices (0.2 mm) of French fries achieved higher overall acceptability compared to thicker slices (0.3 mm, 0.4 mm, and 0.5 mm). This suggests that slice thickness is a critical factor in determining the sensory quality of fried potatoes, as thinner slices tend to be crispier and more evenly cooked, enhancing their sensory attributes [68]. Therefore, the results support the idea that adjusting slice thickness can significantly impact the sensory performance of fried potatoes. 

### 3.4. FTIR, and AFM Characterizations of MIP/SPR

**Figure 1:**
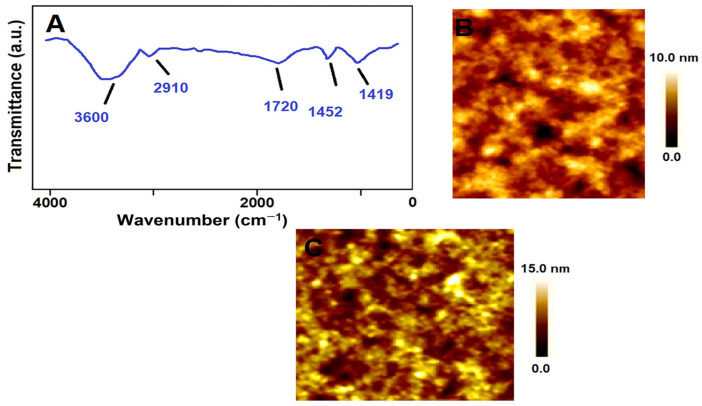
(**A**) FTIR spectra of MIP/SPR chip; AFM images of (**B**) bare SPR chip and (**C**) MIP/SPR chip.

FTIR spectra demonstrated the developed MIP/SPR chip (Figure 1A). The absorption bands at 3600 cm^−1^, 2910 cm^−1^, 1452 cm^−1^, 1720 cm^−1^, and 1419 cm^−1^ corresponded to the –OH stretching mode, MAGA monomer’s saturating –CH stretching, –NH bonding attributing to amide, the carboxyl–carbonyl stretching band, and the –COO– stretching mode, respectively. These results were in alignment with the literature [69]. According to the AFM images, including the bare SPR chip (Figure 1B) and the MIP/SPR chip (Figure 1C), the surface thicknesses were determined as 3.96 ± 0.05 and 20.73 ± 0.06 nm, respectively, verifying AA-imprinted polymer formation on the SPR chip.

### 3.5. pH Effect on the MIP/SPR Chip

**Figure 2:**
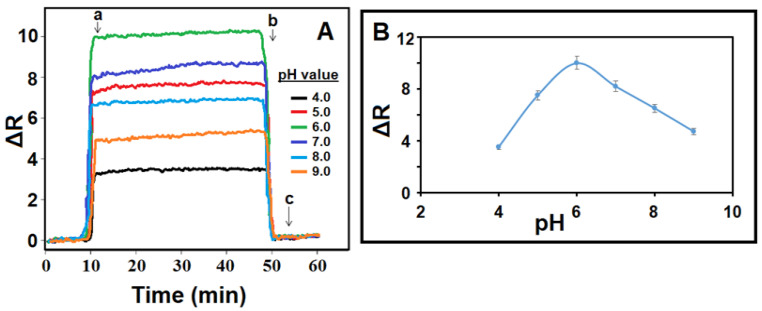
(**A**) SPR sensorgrams for 10.0 nM AA at different pHs of PBS. (**B**) Effect of pH on MIP/SPR chip: (a) adsorption; (b) desorption; (c) regeneration.

Since the degree of light polarization in SPR sensor applications is significantly affected by the method pH, pH optimization is a significant parameter. MAGA, which was preferred as the monomer in this study, is a carboxylic acid-based chemical agent and was used in the anionic phase at low pH values. In such cases, the monomer-analyte interaction was at its maximum, providing the increase in sensor affinity. On the other hand, at high pH values, the sensor affinity decreased owing to the analyte molecule ionization. Hence, pH 6.0 was selected as the optimum pH value for future AA applications (Figure 2A,B) [47]. 

### 3.6. Linearity Range of the MIP/SPR Chip

**Figure 3:**
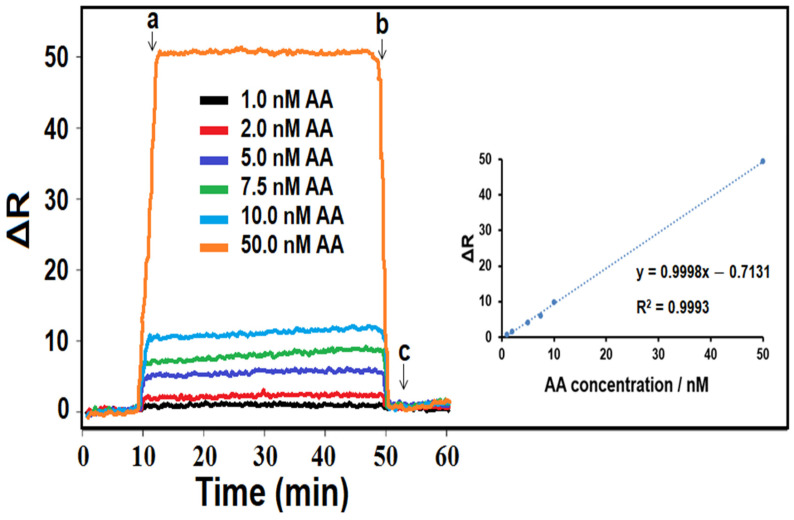
Effect of AA concentration on MIP/SPR chip. Inset: Calibration curve of AA concentrations of MIP/SPR chip in the presence of pH 6.0 of PBS (from 1.0 nM to 50.0 nM AA): (a) adsorption; (b) desorption; (c) regeneration.

SPR is an excellent method for observing changes in the refractive index in the immediate vicinity of a metal surface. SPR sensors are suitable for examining only a limited distance or a fixed volume on the metal surface. In order to perform a selective determination by SPR, the sensor surface can be modified with some modifiers, such as a molecularly imprinted polymer that can selectively recognize the target component. If the sensor surface is properly modified, the target analytes migrate away from the surface while the ligand remains on the surface. It is possible to perform multiple assays using the same sensor chip with a regeneration solution without inhibiting the activity of the ligand [70]. SPR signals were linear in a range from 1.0 to 50.0 nM AA, and the calibration equation of y (ΔR) = 0.9998x (C_AA_, nM) − 0.7131 is shown in Figure 3. The limit of quantification (LOQ) and LOD values were 1.0 × 10^−9^ M and 3.0 × 10^−10^ M, respectively. According to Table 4, the developed SPR sensor performed AA analysis with great sensitivity compared to other techniques presented in the literature. In addition, performing AA analysis instantly and with high selectivity can lead to the prediction of important health problems, such as muscle weakness and sensory and reflex losses.

### 3.7. Recovery of MIP/SPR Chip and AA Content

The SPR sensor, which was prepared for sample analysis and designed to measure the AA level in different-sized fried potato samples (T1, T2, T3, and T4), was applied to the samples. The obtained AA amounts are summarized in Table 5. Moreover, the recovery values close to 100.00% proved that the developed SPR sensor was prepared with high specificity for AA. When examining the mean AA content, it was found that the highest levels were measured in T1 (15.37 ± 0.01 nM) samples, followed by T3 (10.79 ± 0.04 nM), T2 (5.31 ± 0.03 nM), and finally, T4 (3.89 ± 0.08 nM) samples. As observed, the preparation of fried potatoes in different sizes and shapes significantly affected AA formation (*p* < 0.05). In the study, the mean AA content was found to be highest in the T1 fried potato sample, which had dimensions of 50 × 3 mm. The European Commission’s Regulation (EU) 2017/2158 sets benchmark levels for AA in various foods, establishing a benchmark level of 500 μg/kg for ready-to-eat French fries. It was determined that the AA content in the analyzed fried potato samples was below this benchmark level established by the European Food Safety Authority (EFSA). The wide variation in AA formation among individual fried potato samples is directly related to the surface area-to-volume effect and frying conditions [77]. The size and shape of the fried potatoes are critical for AA formation, as it primarily occurs in the surface layer [33,78]. This also supports the differences in AA concentrations observed in our study among differently sliced fried potatoes (Table 3). Consequently, the highest levels of AA were observed in the T1 group, which had a larger surface-to-volume ratio. Similar results were observed by Narvus-Varlı and Mortaş, who applied different preprocessing and cooking techniques to potato slices. The AA concentrations in the potato slices subjected to soaking and cooked by deep frying were found to have a mean of 1.18 μg/kg [79]. In a study conducted by Michalak et al., the mean AA contents of ready-to-eat frozen French fries of different sizes and shapes (crinkle, thick-cut, shoestring, cubes, and wedges) were determined to be 539 ± 45, 568 ± 41, 685 ± 15, 392 ± 18, and 451 ± 19 μg/kg, respectively, with AA concentrations being higher compared to cube-cut fries [33]. In another study, it was reported that the AA concentrations in commercially available fried potato strips ranged between 82.0 and 4245.6 μg/kg [80]. As observed in the literature, the concentration of AA can vary significantly even within the same food samples. These variations may be attributed to several factors, including the variety of potatoes, food composition, the cooking temperature/time, the cooking method, and other variables [61]. Our findings revealed that frying potatoes of different cuts and shapes affects AA levels. However, these results also highlight the importance of developing sensors, such as imprinted SPR, to measure these levels effectively, accurately, and easily. 

### 3.8. Selectivity, Repeatability, and Reusability of MIP/SPR Chip

**Figure 4:**
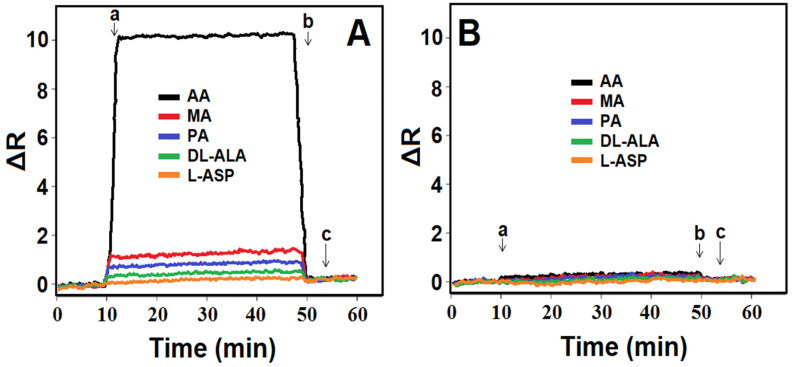
Selectivity tests: SPR sensorgrams of (**A**) MIP/SPR chip and (**B**) NIP/SPR chip in 10.0 nM AA, 1000.0 nM MA, 1000.0 nM PA, 1000.0 nM DL-ALA, 1000.0 nM L-ASP: (a) adsorption; (b) desorption; (c) regeneration.

The high selectivity of the prepared MIP/SPR chip is shown in Figure 4A,B. Selectivity tests were carried out in the presence of MA, PA, DL-ALA, and L-ASP. These chemicals were chosen as competitive agents because they are likely to be found in potato samples together with AA. As anticipated, the SPR sensor designed specifically for AA demonstrated minimal sensitivity to other agents that were chemically similar to AA. In fact, although the concentration of other agents was 100 times higher than the concentration of AA, the developed SPR sensor showed more specificity for AA. This was proof that the molecular imprinting technique provided high selectivity to the target molecule. k and k’ values were greater than 1; hence, the imprinting factor on the SPR chip surface was high (Table 6).

**Figure 5:**
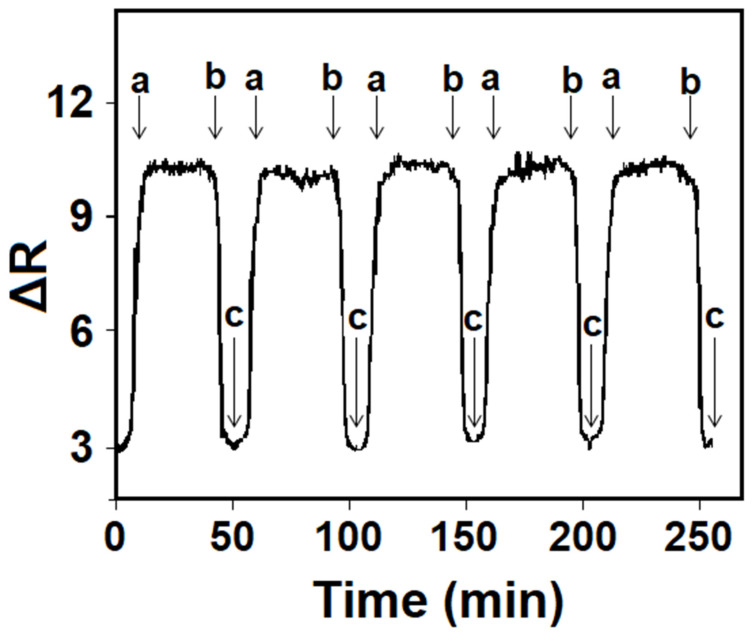
Repeatability of MIP/SPR chip in 10.0 nM AA. (a) adsorption; (b) desorption; (c) regeneration.

For the repeatability test of the developed MIP/SPR chip, five consecutive cycles in the presence of 10.0 nM AA were completed, and the obtained SPR signals for each cycle were almost close to each other with a relative standard deviation of 0.44%, providing high repeatability (Figure 5).

Lastly, for the reusability test of the developed MIP/SPR chip, 30 consecutive SPR signals of a developed MIP/SPR chip were observed in the presence of 10.0 nM AA; SPR signals with a relative standard deviation of 0.21% confirmed the high reusability.

### 3.9. Pearson Correlation Analysis Results

**Figure 6:**
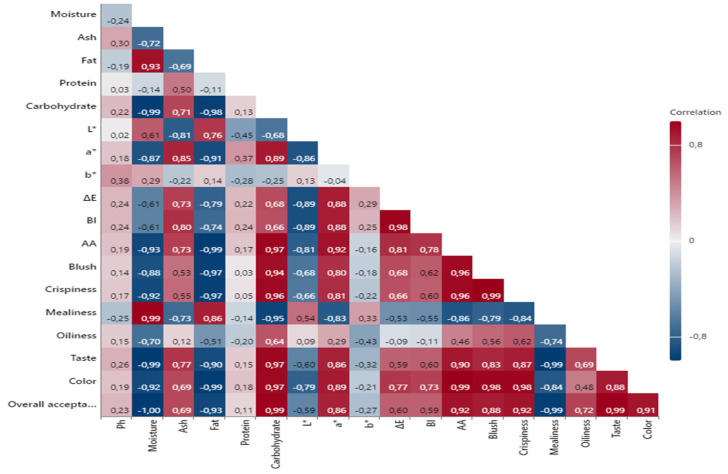
Pearson correlation analysis results. AA: Acrylamide, Overall acceptability: Overall acceptability, Ph: pH.

Figure 6 presents the correlation matrix showing the relationships between the independent variables in our study. Specifically, variables with positive correlations above 0.90 with AA and those with negative correlations below 0.90 were focused on, and it was found that the majority of these correlations were statistically significant (*p* < 0.05). A strong positive correlation was observed between the AA levels in potatoes and the parameters of blush (0.96), crispiness (0.96), color (0.99), taste (0.90), and overall acceptability (0.92). Additionally, when examining the correlation results with color parameters, positive correlations were determined with the a* value (0.92), and strong positive correlations were found with the ΔE (0.81) and BI (0.78) values. Conversely, a negative correlation (−0.81) was found between the L* value and AA levels. In summary, darker (lower L*), redder (higher a*), and browner (higher BI) fried potatoes tended to have higher AA levels. Furthermore, the total color difference (ΔE) was strongly related to AA, indicating that significant color changes during frying were associated with increased AA formation. Conversely, a strong negative correlation was found between the AA levels of fried potatoes of different sizes and their moisture levels (−0.93) and fat contents (−0.99). This indicates that samples with higher moisture and fat contents tended to have lower AA levels. This inverse relationship can be attributed to the fact that higher moisture content may diminish the extent of the Maillard reaction, which is a critical process in AA formation.

In conclusion, the strong correlations between instrumental color parameters (L*, a*, BI) and AA content highlight that color changes and darkening are significant factors affecting AA formation. The association of dark and red hues with higher AA levels indicates that color changes and darkening of the potatoes during frying may increase AA accumulation. Additionally, the relationship between sensory attributes such as crispiness and taste with higher AA levels suggests that the cooking conditions and processing times of the potatoes are closely related to AA formation. These correlation results highlight that controlling AA levels in fried potatoes may have adverse effects on the product’s sensory attributes.

## 4. Conclusions

This study successfully evaluated the AA levels and various quality parameters of homemade fried potatoes prepared in different sizes by integrating principles from the Slow Food Movement with advanced sensor technology. A novel and rapid method utilizing a SPR sensor based on a molecularly imprinted polymer was developed for detecting low levels of AA. Importantly, the results indicate that AA forms at higher levels in thinner and smaller potato slices. This highlights the significant impact of slice size and thickness on AA formation during frying. These findings underscore the importance of carefully considering potato slice dimensions in cooking practices to effectively manage AA levels while maintaining sensory quality. The integration of advanced sensor technology with traditional food preparation principles provides a comprehensive approach to enhancing food safety and quality in homemade fried potatoes.

## Figures and Tables

**Table 1:** Compositional, physical, and chemical parameters of cooked homemade fried potatoes of different sizes.

Parameters	T1	T2	T3	T4
pH	6.31 ± 0.01 ^a^	6.30 ± 0.02 ^a^	6.29 ± 0.01 ^a^	6.29 ± 0.02 ^a^
Moisture (%)	21.84 ± 0.74 ^a^	55.08 ± 0.92 ^b^	47.90 ± 0.29 ^c^	55.31 ± 0.39 ^d^
Protein (%)	6.98 ± 0.06 ^a^	7.01 ± 0.08 ^a^	7.02 ± 0.88 ^a^	6.90 ± 0.40 ^a^
Fat (%)	28.15 ± 0.04 ^c^	30.06 ± 0.51 ^b^	28.96 ± 0.04 ^bc^	30.18 ± 0.08 ^a^
Ash (%)	2.64 ± 0.01 ^a^	2.57 ± 0.09 ^a^	2.50 ± 0.33 ^a^	2.39 ± 0.12 ^a^
Carbohydrate (g/100 g DW)	51.68 ± 0.05 ^a^	11.75 ± 0.13 ^c^	26.14 ± 0.21 ^b^	11.68 ± 0.05 ^c^

^a–d^: Means followed by different lowercase letters in the same row are significantly different. T1: (50 × 3 mm) T2: (20 × 20 × 20 mm), T3: (12 × 12 × 12 mm), T4: (70 × 10 × 10 mm).

**Table 2:** Color parameters of homemade fried potatoes of different sizes.

	L*	a*	b*	ΔE	BI
Fresh potatoes	69.01 ± 0.15	0.38 ± 0.05	21.05 ± 0.08	-	-
T1	51.09 ± 0.12 ^a^	11.78 ± 0.30 ^a^	33.58 ± 0.98 ^a^	24.67 ± 0.73 ^a^	120.36 ± 1.24 ^a^
T2	54.82 ± 0.51 ^b^	7.14 ± 0.30 ^ab^	35.48 ± 0.41 ^a^	21.35 ± 0.16 ^a^	105.39 ± 0.19 ^a^
T3	51.45 ± 0.85 ^a^	8.35 ± 1.12 ^bc^	36.02 ± 2.77 ^a^	24.57 ± 1.44 ^a^	115.40 ± 0.12 ^a^
T4	59.19 ± 1.21 ^c^	3.94 ± 0.42 ^c^	34.45 ± 2.48 ^a^	15.92 ± 1.21 ^b^	81.55 ± 0.17 ^b^

^a–c^: Means followed by different lowercase letters in the same row are significantly different. T1: (50 × 3 mm) T2: (20 × 20 × 20 mm), T3: (12 × 12 × 12 mm), T4: (70 × 10 × 10 mm).

**Table 3:** Sensory assessment results of the homemade fried potatoes.

	Sensory Parameters						
Treatment	Blush (Scores 1–5)	Crispiness (Scores 1–5)	Mealiness (Scores 1–5)	Oiliness (Scores 1–5)	Taste (Scores 1–5)	Color (Scores 1–5)	Overall Acceptability (Scores 1–5)
T1	3.53 ± 0.07 ^a^	4.08 ± 0.07 ^a^	2.68 ± 0.07 ^a^	4.04 ± 0.03 ^a^	4.05 ± 0.01 ^a^	4.02 ± 0.10 ^a^	4.41 ± 0.06 ^a^
T2	2.51 ± 0.02 ^c^	1.91 ± 0.21 ^b^	3.72 ± 0.03 ^a^	2.70 ± 0.01 ^b^	3.13 ± 0.03 ^b^	3.09 ± 0.07 ^b^	2.96 ± 0.04 ^b^
T3	3.24 ± 0.01 ^b^	3.24 ± 0.08 ^a^	3.71 ± 0.06 ^a^	2.87 ± 0.03 ^b^	3.18 ± 0.02 ^b^	3.66 ± 0.04 ^a^	3.29 ± 0.21 ^b^
T4	2.70 ± 0.01 ^c^	2.30 ± 0.06 ^b^	3.78 ± 0.01 ^b^	3.65 ± 0.11 ^a^	3.06 ± 0.14 ^b^	3.04 ± 0.11 ^b^	2.98 ± 0.13 ^b^

^a–c^: Means followed by different lowercase letters in the same row are significantly different (*p* < 0.05). T1: (50 × 3 mm) T2: (20 × 20 × 20 mm), T3: (12 × 12 × 12 mm), T4: (70 × 10 × 10 mm).

**Table 4:** The comparison of the MIP/SPR chip’s performance for AA determination.

Method	Linear Range (M)	LOD (M)	Ref.
Au@Ag NPs SERS sensor	1.0 × 10^−8^ –1.0 × 10^−3^	1.27 × 10^−9^	[71]
Colorimetric aptasensor	1.0 × 10^−8^–1.0 × 10^−4^	1.53 × 10^−6^	[72]
CuNCs/GSH	5.0 × 10^−6^–3.0 × 10^−4^	1.48 × 10^−6^	[73]
CDs/DNA	1.0 × 10^−7^–1.0 × 10^−3^	2.41 × 10^−8^	[74]
PEC-MIP sensor	2.50 × 10^−10^–1.04 × 10^−7^	1.70 × 10^−9^	[75]
Fluorescent sensor based on ssDNA and GelRed	1.0 × 10^−5^–9.5 × 10^−3^	3.0 × 10^−6^	[76]
MIP/SPR chip	1.0 × 10^−9^–5.0 × 10^−8^	3.0 × 10^−10^	This study

**Table 5:** Recovery results of AA (*n* = 6).

Treatment	Added AA (nM)	Found AA (nM)	* Recovery (%)
T1	-	15.37 ± 0.01 ^a^	-
	2.00	17.38 ± 0.02	100.06 ± 0.06
	4.00	19.36 ± 0.06	99.95 ± 0.03
	6.00	21.36 ± 0.08	99.95 ± 0.02
T2	-	5.31 ± 0.03 ^b^	-
	2.00	7.32 ± 0.04	100.14 ± 0.03
	4.00	9.30 ± 0.05	99.89 ± 0.01
	6.00	11.32 ± 0.07	100.09 ± 0.01
T3	-	10.79 ± 0.04 ^c^	-
	2.00	12.78 ± 0.05	99.92 ± 0.07
	4.00	14.80 ± 0.05	100.07 ± 0.09
	6.00	16.80 ± 0.03	100.06 ± 0.04
T4	-	3.89 ± 0.08 ^d^	-
	2.00	5.90 ± 0.02	100.17 ± 0.07
	4.00	6.90 ± 0.07	100.15 ± 0.07
	6.00	9.88 ± 0.03	99.90 ± 0.03

^a–d^: Means followed by different lowercase letters are significantly different (*p* < 0.05). * Recovery = Found AA, nM/Real AA, nM different (*p* < 0.05). T1: (50 × 3 mm) T2: (20 × 20 × 20 mm), T3: (12 × 12 × 12 mm), T4: (70 × 10 × 10 mm).

**Table 6:** k and k′ values of MIP/SPR and NIP/SPR chips (*n* = 6).

	MIP		NIP		
	∆R	k	∆R	k	k′
AA	10.0 ± 0.02	-	0.20 ± 0.03	-	-
MA	1.00 ± 0.01	10.00	0.15 ± 0.01	1.33	7.52
PA	0.75 ± 0.03	13.00	0.10 ± 0.02	2.00	6.50
DL-ALA	0.50 ± 0.06	20.00	0.05 ± 0.01	4.00	5.00
L-ASP	0.25 ± 0.02	40.00	0.01 ± 0.01	20.00	2.00

Analyte concentrations: 10.0 nM AA, 1000.0 nM MA, 1000.0 nM PA, 1000.0 nM DL-ALA, 1000.0 nM L-ASP. k (selectivity coefficient) = ∆R_AA_/∆R_interfering chemical_ and k′ (relative selectivity coefficient) = k_MIP/_k_NIP_.

## Data Availability

The original contributions presented in the study are included in the article, further inquiries can be directed to the corresponding author.

## References

[1] Tokucoglu Yumusak T., Yilmaz K.G., Deligonul S.Z., Cavusgil T. (2024). Slow food and the slow food movement: A case study of consumer activism in Turkiye. IJCHM.

[2] Dimitrovski D., Starčević S., Marinković V. (2024). Which Attributes Are the Most Important in the Context of the Slow Food Festival?. Leis. Sci..

[3] Petrini C. (2013). Slow Food Nation: Why Our Food Should Be Good, Clean, and Fair.

[4] Simonetti L. (2012). The ideology of Slow Food. J. Eur. Stud..

[5] Sobreira É.M.C., Mantovani D., Leocádio Á. (2022). Slow Food as an Alternative Food Consumption: Approaches, principles and product attributes. Res. Soc..

[6] Özer Ç., Bruwier F., Olgay P. (2020). Potatoes with its history, usage in turkish-french cuisine and outstanding. Gastroia J. Gastron. Travel Res..

[7] Çakmak M., Sarıışık M. (2019). An investigation on the basic contents of the main dishes of the Turkish cuisine. Anais Bras. Estud. Tur..

[8] Ciccone M., Chambers D., Chambers E., Talavera M. (2020). Determining Which Cooking Method Provides the Best Sensory Differentiation of Potatoes. Foods.

[9] Ahmed Z.A., Mohammed N.K. (2024). Investigating influencing factors on acrylamide content in fried potatoes and mitigating measures: A review. FPPN.

[10] Bachir N., Akkoum H., Pujola M., Sepulcre F., Haddarah A. (2024). Impact of amino acids and sugars after thermal processing on acrylamide formation in synthetic potato models and real potatoes. Food Sci. Nutr..

[11] Rajesh T.P., Basheer V.A., Packirisamy A.S.B., Ravi S.N., Vallinayagam S. (2024). Effective inhibition of enzymatic browning and carcinogenic acrylamide in fried food by polyphenols. Top Catal..

[12] Perera D.N., Hewavitharana G.G., Navaratne S. (2021). Comprehensive study on the acrylamide content of high thermally processed foods. Biomed Res. Int..

[13] Wenzl T., Lachenmeier D.W., Gökmen V. (2007). Analysis of heat-induced contaminants (acrylamide, chloropropanols and furan) in carbohydrate-rich food. Anal. Bioanal. Chem..

[14] Stadler R.H., Gökmen V. (2024). Acrylamide formation mechanisms. Acrylamide in Food.

[15] Cunha N.M., de Souza Chaves D.H., da Silveira T.M.L., Birchal V.S., Garcia M.A.V.T. (2024). Evaluation of vegetable oil blends properties during deep frying process and their influence on the quality of fried potatoes. OLEL.

[16] Troise A.D., Scaloni A. (2024). Quantitation of acrylamide in foods by high-resolution mass spectrometry. Acrylamide in Food.

[17] Govindaraju I., Sana M., Chakraborty I., Rahman M.H., Biswas R., Mazumder N. (2024). Dietary Acrylamide: A Detailed Review on Formation, Detection, Mitigation, and Its Health Impacts. Foods.

[18] Pan M., Liu K., Yang J., Hong L., Xie X., Wang S. (2020). Review of Research into the Determination of Acrylamide in Foods. Foods.

[19] Wang P., Ji R., Ji J., Chen F. (2019). Changes of metabolites of acrylamide and glycidamide in acrylamide-exposed rats pretreated with blueberry anthocyanins extract. Food Chem..

[20] Halford N.G., Curtis T.Y., Muttucumaru N., Postles J., Elmore J.S., Mottram D.S. (2012). The acrylamide problem: A plant and agronomic science issue. J. Exp. Bot..

[21] Rifai L., Saleh F.A. (2020). A review on acrylamide in food: Occurrence, toxicity, and mitigation strategies. IJT.

[22] Abedini A.H., Vakili Saatloo N., Salimi M., Sadighara P., Alizadeh Sani M., Garcia-Oliviera P., Prieto M.A., Kharazmi M.S., Jafari S.M. (2024). The role of additives on acrylamide formation in food products: A systematic review. Crit. Rev. Food Sci. Nutr..

[23] Hamzalıoğlu A., Gökmen V. (2020). 5-Hydroxymethylfurfural accumulation plays a critical role on acrylamide formation in coffee during roasting as confirmed by multiresponse kinetic modelling. Food Chem..

[24] Corrêa C.L.O., das Merces Penha E., Dos Anjos M.R., Pacheco S., Freitas-Silva O., Luna A.S., Gottschalk L.M.F. (2021). Use of asparaginase for acrylamide mitigation in coffee and its influence on the content of caffeine, chlorogenic acid, and caffeic acid. Food Chem..

[25] Başaran B., Çuvalcı B., Kaban G. (2023). Dietary acrylamide exposure and cancer risk: A systematic approach to human epidemiological studies. Foods.

[26] Jozinović A., Panak Balentić J., Ačkar Đ., Benšić M., Babić J., Barišić V., Lončarić A., Miličević B., Šubarić D. (2024). Nutritionally Valuable Components and Heat-Induced Contaminants in Extruded Snack Products Enriched with Defatted Press Cakes. Molecules.

[27] Monien B.H., Bergau N., Gauch F., Weikert C., Abraham K. (2024). Internal exposure to heat-induced food contaminants in omnivores, vegans and strict raw food eaters: Biomarkers of exposure to acrylamide (hemoglobin adducts, urinary mercapturic acids) and new insights on its endogenous formation. Arch. Toxicol..

[28] Cascos G., Montero-Fernández I., Marcía-Fuentes J.A., Aleman R.S., Ruiz-Canales A., Martín-Vertedor D. (2024). Electronic Prediction of Chemical Contaminants in Aroma of Brewed Roasted Coffee and Quantification of Acrylamide Levels. Foods.

[29] EFSA Panel on Contaminants in the Food Chain (CONTAM) (2015). Scientific opinion on acrylamide in food. EFSA J..

[30] Başaran B., Aydın F., Kaban G. (2020). The determination of acrylamide content in brewed coffee samples marketed in Turkey. Food Addit. Contam. Part A.

[31] Mesias M., Delgado-Andrade C., Morales F.J. (2022). An updated view of acrylamide in cereal products. Curr. Opin. Food Sci..

[32] Dağoğlu I., Keskin Alkaç Z., Korkak F.A., Kazdal S.M., Dağ A. (2024). Acrylamide in heat-treated carbohydrate-rich foods in Turkey. Food Addit. Contam. Part B.

[33] Michalak J., Gujska E., Klepacka J. (2011). The effect of domestic preparation of some potato products on acrylamide content. Plant Foods Hum. Nutr..

[34] Foot R., Haase N.U., Grob K., Gonde P. (2007). Acrylamide in fried and roasted potato products: A review on progress in mitigation. Food Addit. Contam. Part A.

[35] Bethke P.C., Bussan A.J. (2013). Acrylamide in processed potato products. AJPR.

[36] Gielecińska I., Mojska H. (2023). Trends in the acrylamide content of potato products in Poland in the years 2004–2020. Food Control.

[37] Krishnakumar T., Visvanathan R. (2014). Acrylamide in food products: A review. J. Food Process Technol..

[38] Mihai A.L., Negoiță M., Horneț G.A. (2022). Assessment of acrylamide in potato chips and French fries consumed by the Romanian population. JFPP.

[39] Sharafi K., Kiani A., Massahi T., Mansouri B., Ebrahimzadeh G., Moradi M., Fattahi N., Omer A.K. (2024). Acrylamide in potato chips in Iran, health risk assessment and mitigation. Food Addit. Contam. Part B.

[40] Shishov A., Markova U., Ekaterina D., Bulatov A. (2024). Automated liquid–liquid deep eutectic solvents based microextraction procedure for determination of acrylamide in foodstuffs by high-performance liquid chromatography with ultraviolet detection. JFST.

[41] Omar M.M.A., Elbashir A.A., Schmitz O.J., Ziyada A.K., Osman A. (2024). Validation of high-performance liquid chromatography coupled with LTQ orbitrap mass spectrometry for analysis of acrylamide. J. Food Meas. Charact..

[42] Vaisocherová-Lísalová H., Víšová I., Ermini M.L., Špringer T., Song X.C., Mrázek J., Lamačová J., Lynn N.S., Šedivák P., Homola J. (2016). Low-fouling surface plasmon resonance biosensor for multi-step detection of foodborne bacterial pathogens in complex food samples. Biosens. Bioelectron..

[43] Atar N., Eren T., Yola M.L. (2015). A molecular imprinted SPR biosensor for sensitive determination of citrinin in red yeast rice. Food Chem..

[44] Akıcı Ş.Y., Bankoğlu Yola B., Karslıoğlu B., Polat İ., Atar N., Yola M.L. (2023). Fenpicoxamid-imprinted surface plasmon resonance (SPR) sensor based on sulfur-doped graphitic carbon nitride and its application to rice samples. Micromachines.

[45] Li F., Yue S., Zhao Z., Liu K., Wang P., Zhan S. (2024). Application of molecularly imprinted polymers in the water environmental field: A review on the detection and efficient removal of emerging contaminants. Mater Today Sustain..

[46] Blackburn C., Sullivan M.V., Wild M.I., O’Connor A.J., Turner N.W. (2024). Utilisation of molecularly imprinting technology for the detection of glucocorticoids for a point of care surface plasmon resonance (SPR) device. Anal. Chim. Acta.

[47] Capar N., Yola B.B., Polat İ., Bekerecioğlu S., Atar N., Yola M.L. (2023). A zearalenone detection based on molecularly imprinted surface plasmon resonance sensor including sulfur-doped g-C3N4/Bi2S3 nanocomposite. Microchem. J..

[48] Liu Y., Wang L., Li H., Zhao L., Ma Y., Zhang Y., Liu J., Wei Y. (2024). Rigorous recognition mode analysis of molecularly imprinted polymers—Rational design, challenges, and opportunities. Prog. Polym. Sci..

[49] Wang L., Pagett M., Zhang W. (2023). Molecularly imprinted polymer (MIP) based electrochemical sensors and their recent advances in health applications. Sens. Actuators Rep..

[50] Gamal M., Abdelwahab N.S., Imam M.S., Albugami A.S., Hunjur S.A., Aldhalmi A.K., AbdElrahman M., Ghoneim M.M., Ali H.M., Eissa M.S. (2024). Current advances in the implementation of magnetic molecularly imprinted polymers tailored for enrichment of target analytes in different environmental samples: An overview from a comprehensive perspective. Trends Environ. Anal..

[51] Li Y., Luo L., Kong Y., Li Y., Wang Q., Wang M., Li Y., Davenport A., Li B. (2024). Recent advances in molecularly imprinted polymer-based electrochemical sensors. Biosens. Bioelectron..

[52] Zhao D., Zhang Y., Ji S., Lu Y., Bai X., Yin M., Huang C., Jia N. (2021). Molecularly imprinted photoelectrochemical sensing based on ZnO/polypyrrole nanocomposites for acrylamide detection. Biosens. Bioelectron..

[53] Zhang C., Shi X., Yu F., Quan Y. (2020). Preparation of dummy molecularly imprinted polymers based on dextran-modified magnetic nanoparticles Fe3O4 for the selective detection of acrylamide in potato chips. Food Chem..

[54] Verma V., Yadav N. (2022). Acrylamide content in starch based commercial foods by using high performance liquid chromatography and its association with browning index. CRFS.

[55] International A. (2000). Official Methods of Analysis.

[56] Nateghi L., Hosseini E., Fakheri M.A. (2024). The effect of cold atmospheric plasma pretreatment on oil absorption, acrylamide content and sensory characteristics of deep-fried potato strips. Food Chem. X.

[57] Alimi B.A., Shittu T.A., Sanni L.O., Arowolo T. (2013). Effect of pre-drying and hydrocolloid type on colour and textural properties of coated fried yam chips. NIFOJ.

[58] Jaggan M., Mu T., Sun H. (2020). The effect of potato (*Solanum tuberosum* L.) cultivars on the sensory, nutritional, functional, and safety properties of French fries. JFPP.

[59] Pedreschi F. (2012). Frying of potatoes: Physical, chemical, and microstructural changes. Dry.Technol..

[60] Arisseto A.P., Silva W.C., Marcolino P.F.C., Scaranelo G.R., Berbari S.A.G., de Oliveira Miguel A.M.R., Vicente E. (2019). Influence of potato cultivar, frying oil and sample pre-treatments on the contamination of French fries by 3-monochloropropane-1, 2-diol fatty acid esters. Int. Food Res..

[61] Adimas M.A., Abera B.D., Adimas Z.T., Woldemariam H.W., Delele M.A. (2024). Traditional food processing and Acrylamide formation: A review. Heliyon.

[62] Vaitkevičienė N., Jarienė E., Kulaitienė J., Levickienė D. (2022). The physico-chemical and sensory characteristics of coloured-flesh potato chips: Influence of cultivar, slice thickness and frying temperature. Appl. Sci..

[63] Pedreschi F., Kaack K., Granby K. (2006). Acrylamide content and color development in fried potato strips. Int. Food Res..

[64] Tajner-Czopek A., Figiel A., Carbonell-Barrachina A.A. (2008). Effects of potato strip size and pre-drying method on French fries quality. Eur. Food Res. Technol..

[65] Dite Hunjek D., Pranjić T., Repajić M., Levaj B. (2020). Fresh-cut potato quality and sensory: Effect of cultivar, age, processing, and cooking during storage. J. Food Sci..

[66] Salvador A., Varela P., Sanz T., Fiszman S. (2009). Understanding potato chips crispy texture by simultaneous fracture and acoustic measurements, and sensory analysis. LWT Food Sci. Technol..

[67] Abong G.O., Okoth M.W., Imungi J.K., Kabira J.N. (2011). Effect of slice thickness and frying temperature on color, texture and sensory properties of crisps made from four kenyan potato cultivars. Am. J. Food Technol..

[68] Kaundal B., Sharma V., Bansal D., Khagwal A., Singh B. (2022). Comparative Study of Frying to Different Slice Thickness of Potato: Effect on Nutritive Value. Braz. Arch. Biol. Technol..

[69] Yola M.L., Uzun L., Özaltın N., Denizli A. (2014). Development of molecular imprinted nanosensor for determination of tobramycin in pharmaceuticals and foods. Talanta.

[70] Homola J. (2008). Surface plasmon resonance sensors for detection of chemical and biological species. Chem. Rev..

[71] Wang H., Zhang L., Chen C., Waterhouse G.I., Sun Y., Xu Z. (2024). SERS Sensor Based on Core–Shell Au@ Ag Nanoparticles for the Sensitive Detection of Acrylamide in Foods. Food Anal. Methods.

[72] Guo K., Lin X., Duan N., Lu C., Wang Z., Wu S. (2024). Detection of acrylamide in food based on MIL-glucose oxidase cascade colorimetric aptasensor. Anal. Chim. Acta.

[73] Cheng B., Xia X., Han Z., Yu H., Xie Y., Guo Y., Yao W., Qian H., Cheng Y. (2024). A ratiometric fluorescent “off-on” sensor for acrylamide detection in toast based on red-emitting copper nanoclusters stabilized by bovine serum albumin. Food Chem..

[74] Wei Q., Zhang P., Liu T., Pu H., Sun D.-W. (2021). A fluorescence biosensor based on single-stranded DNA and carbon quantum dots for acrylamide detection. Food Chem..

[75] Zhang C., Ou W., Zeng Z., Liu H., Yu K., Wang L., Zhou L. (2024). Polypyrrole participates in the construction of a polarity-switchable photoelectrochemical molecularly imprinted sensor for the detection of acrylamide in fried foods. Sens. Actuators B Chem..

[76] Asnaashari M., Kenari R.E., Taghdisi S.M., Abnous K., Farahmandfar R. (2023). A novel fluorescent DNA sensor for acrylamide detection in food samples based on single-stranded DNA and GelRed. J. Fluoresc..

[77] Gökmen V., Palazoğlu T.K., Şenyuva H.Z. (2006). Relation between the acrylamide formation and time–temperature history of surface and core regions of French fries. J. Food Eng..

[78] Norén L. (2019). Acrylamide in Ready-to-Eat Potato Products. Master’s Thesis.

[79] Navruz-Varlı S., Mortaş H. (2024). Acrylamide formation in air-fried versus deep and oven-fried potatoes. Front. Nutr..

[80] Shamla L., Nisha P. (2014). Acrylamide in deep-fried snacks of India. Food Addit. Contam. Part B.

